# Restoration of regulatory B cell deficiency following alemtuzumab therapy in patients with relapsing multiple sclerosis

**DOI:** 10.1186/s12974-018-1334-y

**Published:** 2018-10-30

**Authors:** Yeseul Kim, Gayoung Kim, Hyun-June Shin, Jae-Won Hyun, Su-Hyun Kim, Eunjig Lee, Ho Jin Kim

**Affiliations:** 1 Division of Clinical Research, Research institute, National Cancer Center, Goyang, Korea; 2 Department of Neurology, Research Institute and Hospital of National Cancer Center, Goyang, Korea; 3 Yonsei University College of Medicine, Seoul, Korea

**Keywords:** Multiple sclerosis, Regulatory B cells, Alemtuzumab, Relapse

## Abstract

**Background:**

Regulatory B cells (Bregs), which protect from autoimmunity, are deficient in multiple sclerosis (MS). Novel regulatory B cell subsets CD19^+^CD24^hi^CD38^hi^ cells and CD19^+^PD-L1^hi^ cells, with disparate regulatory mechanisms have been defined. Alemtuzumab provides a long-lasting suppression of disease activity in MS. In contrast to its documented efficacy, alemtuzumab’s mechanism of action is not fully understood and information about the composition of repopulating B cell pool is scarce.

**Aim:**

To characterize repopulated B cell subsets and elucidate alemtuzumab’s mechanism of action in B cell perspective.

**Methods:**

The frequency and the absolute number of Bregs were studied in peripheral blood mononuclear cells (PBMC) of 37 MS patients and 11 healthy controls (HC). Longitudinal analysis of the frequency and the absolute number of Bregs in PBMC of 11 MS patients was evaluated, before and at 6, 9, and 12 months post alemtuzumab.

**Results:**

We found deficiency of CD19^+^CD24^hi^CD38^hi^ cells during relapse compared to remission and HC (relapse vs remission: *p* = 0.0006, relapse vs HC: *p* = 0.0004). CD19^+^PD-L1^hi^ cells were deficient during relapse than remission and HC (relapse vs remission: *p* = 0.0113, relapse vs HC: *p* = 0.0007). Following alemtuzumab, the distribution of B cells shifts towards naïve phenotype and Breg deficiency is restored. The frequency of CD19^+^CD24^hi^CD38^hi^ cells was significantly increased at 6 M and 9 M compared to 0 M (6 M vs 0 M: *p* = 0.0004, 9 M vs 0 M: *p* = 0.0079). At 9 M, the frequency of CD19^+^CD24^hi^CD38^hi^ cells started to decrease and by 12 M the frequency was reduced compared to 6 M, although it was significantly higher than baseline level (12 M vs 0 M: *p* = 0.0257). The absolute number was significantly increased at 6 M and 9 M post-alemtuzumab (6 M vs 0 M: *p* = 0.0063, 9 M vs 0 M: *p* = 0.02). The frequency of CD19^+^PD-L1^hi^ cells significantly increased until 12 M (6 M vs 0 M: *p* = 0.0004, 12 M vs 0 M: *p* = 0.0036). The frequency of CD19^+^PD-L1^hi^ cells at 12 M was significantly higher than 9 M (*p* = 0.0311). We further pinpoint that CD19^+^CD24^hi^CD38^hi^ cells were deficient at severe relapses following alemtuzumab infusion and restored during recovery.

**Conclusions:**

Our results highlight the preferential reconstitution of Bregs as a possible mechanism of action of alemtuzumab and CD19^+^CD24^hi^CD38^hi^ cells as a potential biomarker for disease activity.

## Background

The concept that multiple sclerosis (MS) is a T-cell-mediated disease has been changed and it is now widely accepted that B cells play a part in the pathogenesis of MS. [1–3] Multiple roles of B cells have been elucidated emphasizing that B cells have dual contribution to autoimmunity [1, 4–6]. Hence, the concept of regulatory B cells (Breg) has emerged, proving that B cell subset distribution is far more complex than the original concept. A novel Breg subset that was originally known as immature transitional B cells (CD19^+^CD24^hi^CD38^hi^) has been described to have regulatory capacity through interleukin-10 production [7]. Another study has found B cells highly expressing programmed death ligand-1 (CD19^+^PD-L1^hi^ cells) that exert regulatory function through cell-to-cell contact via interaction of CD19^+^PD-L1^hi^ cells with PD-1 on T cells, resulting in suppression of T follicular helper (Tfh) cell differentiation and expansion, which are the cell type known to be involved in the relapse of MS patients [8, 9].

Alemtuzumab is a highly effective treatment in relapsing MS. It provides a long-lasting suppression of disease activity by altering the proportion of lymphocyte subsets with preferential increase of regulatory T cells (Treg) [10, 11]. In contrast to alemtuzumab’s documented efficacy, alemtuzumab’s mechanism of action is not fully understood and information about the composition of the repopulating B cell pool, especially Breg, is scarce.

Here, we pinpoint deficiency of CD19^+^CD24^hi^CD38^hi^ and CD19^+^PD-L1^hi^ cells during relapse and subsequent expansion following alemtuzumab infusion. We also highlight the possible clinical implication of CD19^+^CD24^hi^CD38^hi^ cells.

## Methods

### Study population

For cross-sectional study, 20 MS patients during relapse (MS-relapse) and 17 MS patients in remission (MS-remission) and 11 healthy controls (HC) were included. For longitudinal analysis, 11 patients who were treated with alemtuzumab were included. All MS patients fulfilled the 2010 McDonald’s criteria [12]. Current study was approved by the Institutional Review Board, and informed consent was obtained from each subject. Demographic and clinical characteristics of participants are summarized in Table 1.

**Table 1:** Demographic and clinical characteristics of MS patients in relapse and remission

Characteristic	MS relapse	MS remission
Age (years, mean ± SD)	34.5 ± 9.4	36.9 ± 5.2
Women:men (*n*:*n*)	16:4	10:7
Onset age (years, mean ± SD)	30.4 ± 22.0	29.8 ± 7.3
Disease duration (years, mean ± SD)	12 ± 24.9	6 ± 5.9
EDSS (mean ± SD)	2.9 ± 1.9	1.8 ± 1.9

### Flow cytometry

For cross-sectional study, fresh peripheral blood mononuclear cells (PBMCs) were surface stained with monoclonal antibodies against CD19-APC-cy7, CD27-FITC, CD24-BV421, CD38-BV510, and PD-L1(CD274)-PE-cy7 (BD Biosciences). For longitudinal study, frozen PBMCs, collected from 11 RRMS patients undergoing alemtuzumab at baseline and 6, 9, and 12 months, were surface stained with monoclonal antibodies as stated above.

### Statistical analysis

We performed analysis of significance in Prism (GraphPad, La Jolla, USA). For cross-sectional data, one-way ANOVA analysis with Tukey’s multiple comparison post hoc analysis was performed to compare the frequency of Bregs between HC, MS-relapse, and MS-remission. Unpaired *t* test was used to compare the absolute number of Bregs between MS-relapse and MS-remission. Repeated measures ANOVA with Tukey’s multiple comparison post hoc analysis was performed for longitudinal analysis. A *p* value of < 0.05 was considered statistically significant. All values show mean ± SEM.

## Result

### Regulatory B cells are deficient in MS patients during relapse

**Fig. 1:**
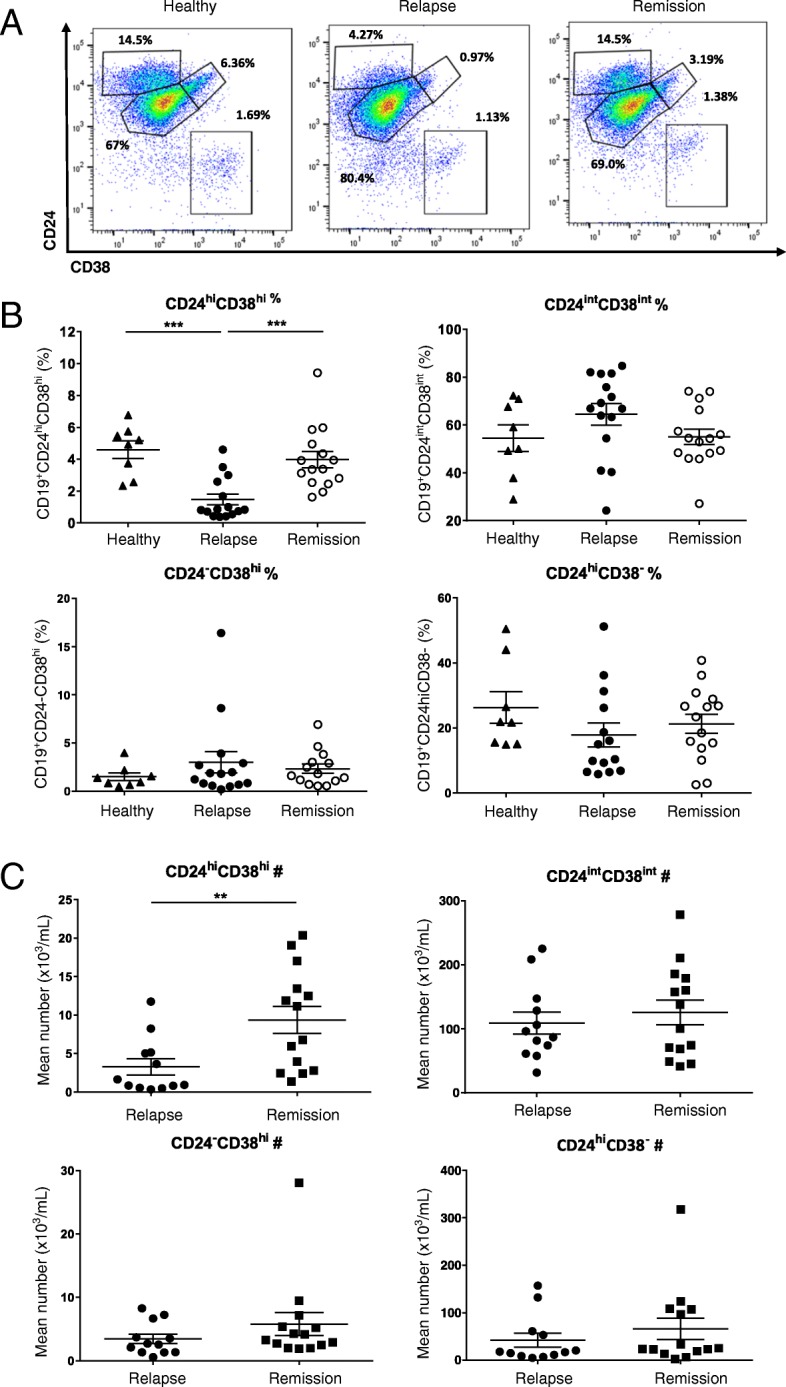
MS patients show deficiency of CD19^+^CD24^hi^CD38^hi^ cells during relapse. The percentage and the absolute number of CD19^+^CD24^hi^CD38^hi^ B cells were measured in MS patients undergoing relapse (*n* = 15) and patients in remission (*n* = 15) and healthy controls (*n* = 8). PBMC was isolated from fresh peripheral blood and surface stained for flow cytometry. **a** Representative flow cytometry dot plot of CD24 and CD38 expression in total CD19+ B cells. **b** Scatter plots showing the percentage of CD19^+^CD24^hi^CD38^hi^ B cells in MS-relapse, MS-remission, and HC. A significant reduction in the frequency of CD19^+^CD24^hi^CD38^hi^ B cells was observed in MS-relapse compared to MS-remission and HC (relapse vs remission: *p* = 0.0006, relapse vs healthy: *p* = 0.0004). All values show mean ± SEM. Data were analyzed by one-way analysis of variance (ANOVA) with Tukey’s multiple comparison post hoc analysis. ***p < 0.001. **c** The absolute number of CD19^+^CD24^hi^CD38^hi^ B cells in MS-relapse was significantly reduced compared to MS-remission (*p* = 0.009). All values show mean ± SEM. Data were analyzed by unpaired *t* test. **p < 0.01. Ex vivo data were collected from peripheral blood samples taken during the time course of this study

**Fig. 2:**
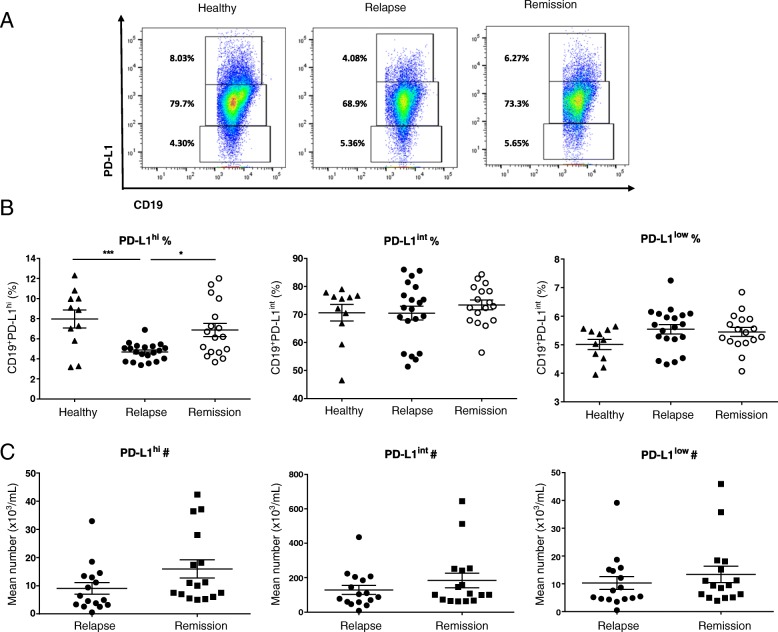
MS patients have deficiency of CD19^+^PD-L1^hi^ cells during relapse. The percentage and the absolute number of CD19^+^PD-L1^hi^ cells were measured in MS patients undergoing relapse (*n* = 20) and patients in remission (*n* = 17) and healthy controls (*n* = 11). Fresh PBMC was isolated from peripheral blood and surface stained for flow cytometry. **a** Representative flow-cytometry dot plot of PD-L1 and CD19 expression in total CD19+ B cells. **b** Scatter plots showing the percentage of CD19^+^PD-L1^hi^ cells in MS-relapse, MS-remission, and HC. The frequency of CD19^+^PD-L1^hi^ cells was significantly reduced in MS-relapse compared to MS-remission and HC (relapse vs remission: *p* = 0.0113, relapse vs healthy: *p* = 0.0007). All values show mean ± SEM. Data were analyzed by one-way analysis of variance (ANOVA) with Tukey’s multiple comparison post hoc analysis. *p < 0.05; ***p < 0.001. **c** Scatter plots showing the absolute number of CD19^+^PD-L1^hi^ cells in MS-relapse and MS-remission. All values show mean ± SEM. Data were analyzed by unpaired *t* test. Ex vivo data were collected from peripheral blood samples taken during the time course of this study

In order to evaluate the relationship between the frequency of CD19^+^CD24^hi^CD38^hi^ cells and CD19^+^PD-L1^hi^ cells with disease activity of MS, the frequency and the absolute number were measured in total CD19^+^ B cells in MS-relapse and MS-remission and HC. The frequency of both CD19^+^CD24^hi^CD38^hi^ cells (Fig. 1a, b) and CD19^+^PD-L1^hi^ cells (Fig. 2a, b) was significantly reduced in MS-relapse compared to MS-remission and HC. The average frequency of Bregs in MS-remission was lower than those of HC, but no statistical difference was observed. The absolute number of CD19^+^CD24^hi^CD38^hi^ cells was significantly reduced in MS-relapse compared to MS-remission (Fig. 1c). Although no significant difference was observed, the absolute number of CD19^+^PD-L1^hi^ cells was reduced in MS-relapse compared to MS-remission (Fig. 2c).

### Preferential reconstitution of naïve B cells following alemtuzumab

**Fig. 3:**
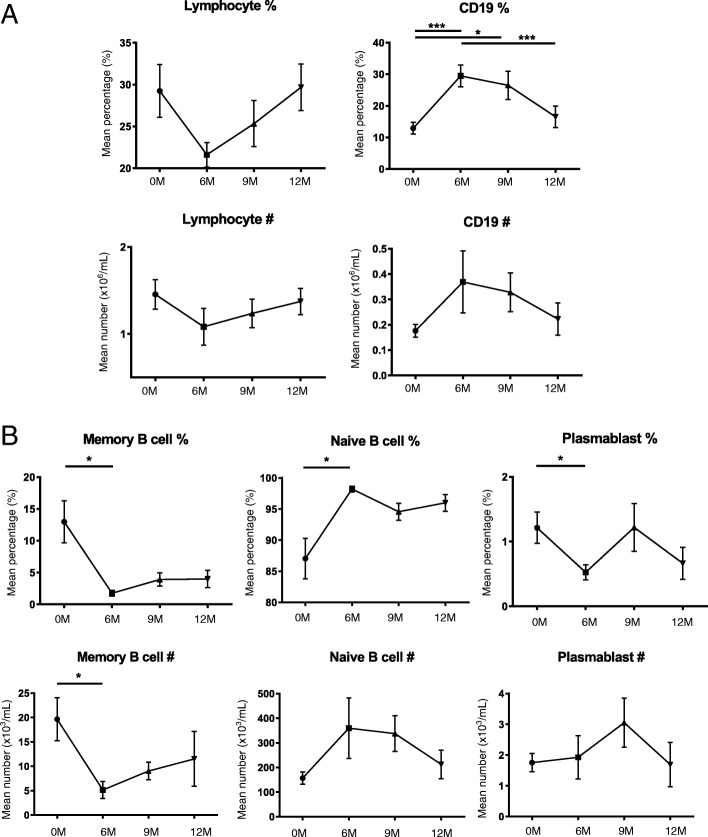
Naive B cells predominate repopulated CD19+ cells following alemtuzumab treatment. In order to evaluate the B cell subset distribution post-alemtuzuamb, thawed PBMCs of alemtuzumab-treated patients (*n* = 11) were evaluated up to 12 months after induction. **a** Cumulative data for the frequency and the absolute number of total lymphocytes and CD19^+^ B cells. Successful depletion and reconstitution of lymphocytes and CD19^+^ B cells was confirmed. **b** Cumulative data for the frequency and the absolute number of CD19^+^CD27^+^ memory B cells, CD19^+^CD27^−^ naïve B cells, and CD19^+^CD27^+^CD38^hi^ plasmablasts. Following alemtuzumab, significant reduction in the frequency of memory B cells (6 M vs 0 M: *p* = 0.0278) and plasmablasts (6 M vs 0 M: *p* = 0.0448) was observed and dominance of naïve B cells was observed (6 M vs 0 M: *p* = 0.0269). The absolute number of memory B cells was significantly decreased compared to 0 M (6 M vs 0 M: *p* = 0.0112). All values show mean ± SEM. Data were analyzed by repeated measures ANOVA with Tukey’s multiple comparison post hoc analysis

As expected, the frequency and absolute number of total lymphocytes was decreased at 6 months and gradually increased up to 12 months post alemtuzumab (Fig. 3a). The frequency and the absolute number of memory B cells and plasmablasts were significantly decreased compared to pre-treatment level, and naïve B cells comprised the majority of repopulated CD19^+^ B cells (Fig. 3b).

### Breg deficiency in MS is restored following alemtuzumab

**Fig. 4:**
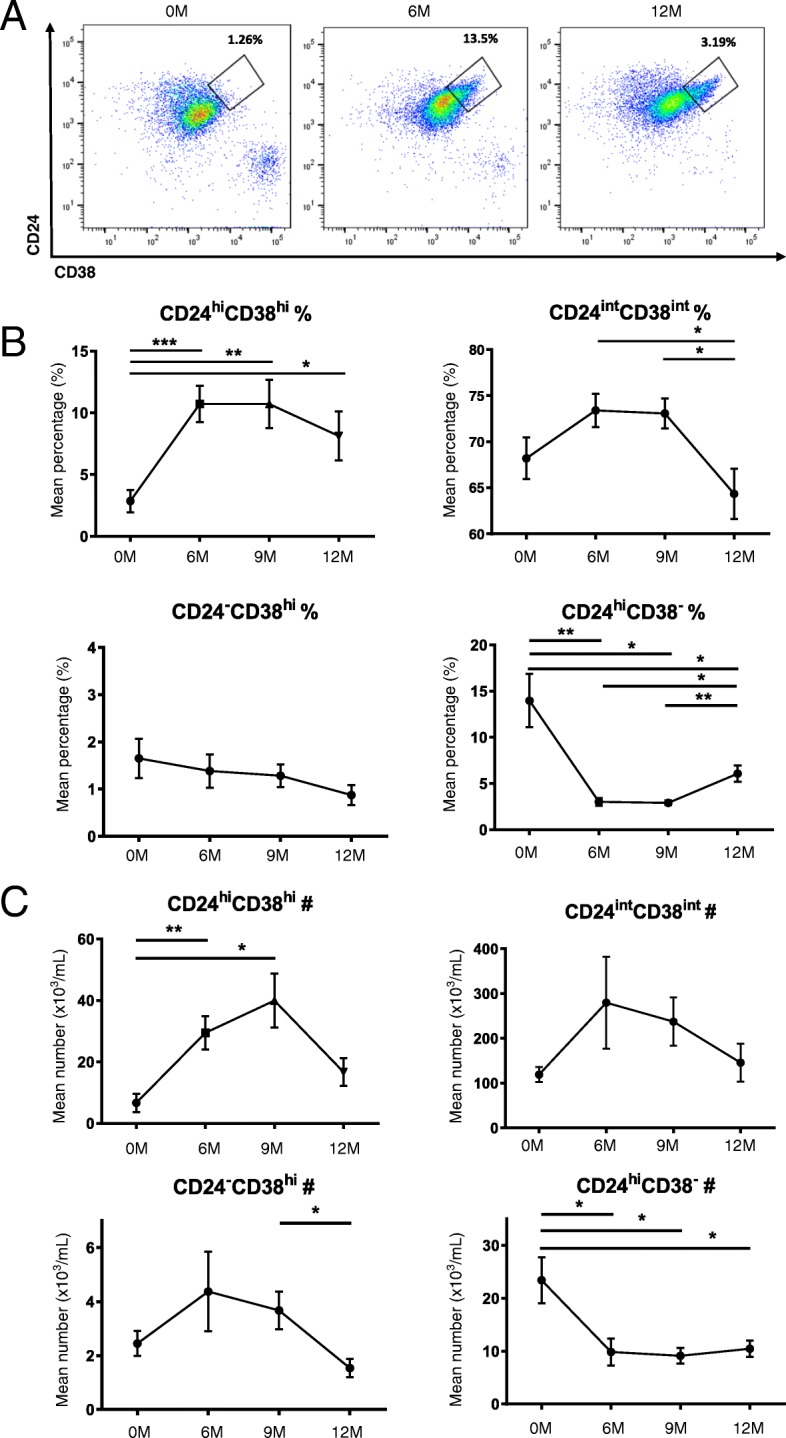
Alemtuzumab treatment restores CD19^+^CD24^hi^CD38^hi^ cells. In order to evaluate the B cell subset distribution post-alemtuzuamb, thawed PBMCs of alemtuzumab-treated patients (*n* = 11) were evaluated up to 12 months after induction. **a** Representative flow-cytometry dot plot of CD24 and CD38 in total CD19^+^ B cells. **b**. Cumulative data for the frequency of CD19^+^CD24^hi^CD38^hi^ B cells. The frequency of CD19^+^CD24^hi^CD38^hi^ cells were significantly increased at 6 M and 9 M compared to pre-treatment level (6 M vs 0 M: *p* = 0.0004, 9 M vs 0 M: *p* = 0.0079). At 9 M, the frequency of CD19^+^CD24^hi^CD38^hi^ cells started to decrease and by 12 M, the frequency was reduced compared to 6 M, although it was significantly increased than baseline level (12 M vs 0 M: *p* = 0.0257). **c** The absolute number was significantly increased at 6 M and 9 M post-alemtuzumab (6 M vs 0 M: *p* = 0.0063, 9 M vs 0 M: *p* = 0.02). All values show mean ± SEM. Data were analyzed by repeated measures ANOVA with Tukey’s multiple comparison post-hoc analysis. *p < 0.05; **p < 0.01; ***p < 0.001

The frequency and the absolute number of CD19^+^CD24^hi^CD38^hi^ cells were markedly increased at 6 and 9 months following alemtuzumab treatment compared to pre-treatment level. By the end of the cycle (12 M), both the frequency and number were decreased, although did not reach pre-treatment level. The frequency and absolute number of CD19^+^CD24^int^CD38^int^ mature naïve B cells were increased at 6 and 9 months post-alemtuzumab, and at 12 months post-alemtuzumab, the frequency of CD19^+^CD24^int^CD38^int^ cells was lower than baseline level. A significant decrease in the frequency and absolute number of CD19^+^CD24^hi^CD38^−^ memory B cells was observed following alemtuzumab treatment (Fig. 4a–c).

**Fig. 5:**
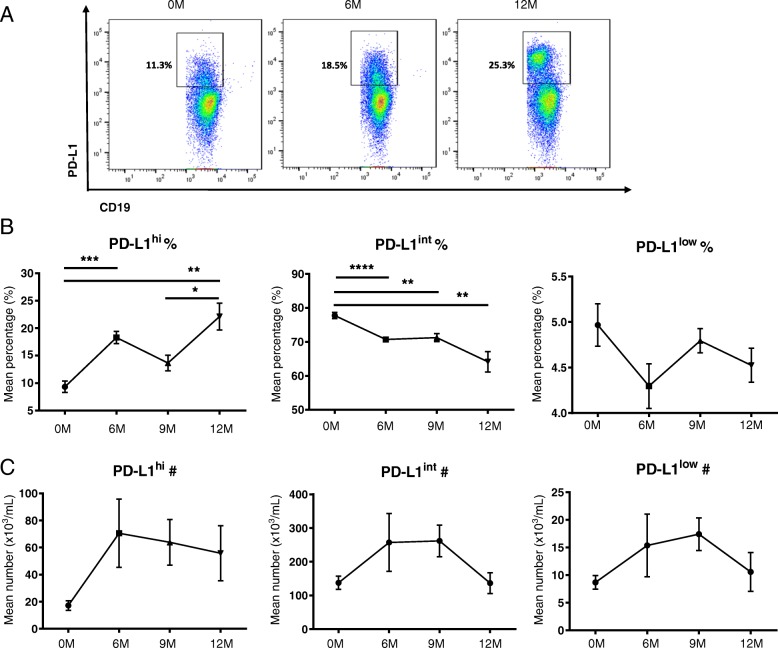
Alemtuzumab treatment restores CD19^+^PD-L1^hi^ cells. In order to evaluate the B cell subset distribution post-alemtuzuamb, thawed PBMCs of alemtuzumab-treated patients (*n* = 11) were evaluated up to 12 months after induction. **a** Representative flow-cytometry dot plot of PD-L1 in total CD19^+^ B cells. **b** Cumulative data for the frequency of CD19^+^PD-L1^hi^ cells. The frequency of CD19^+^PD-L1^hi^ cells increased significantly until 12 M (6 M vs 0 M: *p* = 0.0004, 12 M vs 0 M: *p* = 0.0036). **c** Cumulative data for the absolute number of CD19^+^PD-L1^hi^ cells. No significant difference was observed in the absolute number of CD19^+^PD-L1^hi^, CD19^+^PD-L1^int^, and CD19^+^PD-L1^low^ cells. All values show mean ± SEM. All values show mean ± SEM. Data were analyzed by repeated measures ANOVA with Tukey’s multiple comparison post hoc analysis.*p < 0.05; **p < 0.01; ***p < 0.001; ****p < 0.0001

The frequency and absolute number of CD19^+^PD-L1^hi^ cells were increased at 6 and 9 months and maintained until the end of the cycle. The frequency of CD19^+^PD-L1^int^ cells was significantly decreased at 6, 9, and 12 months following alemtuzumab. The absolute number of CD19^+^PD-L1^int^ cells showed no significant difference. The frequency and absolute number of CD19^+^PD-L1^low^ cells showed no significant difference (Fig. 5a–c).

### Clinical implications

**Fig. 6:**
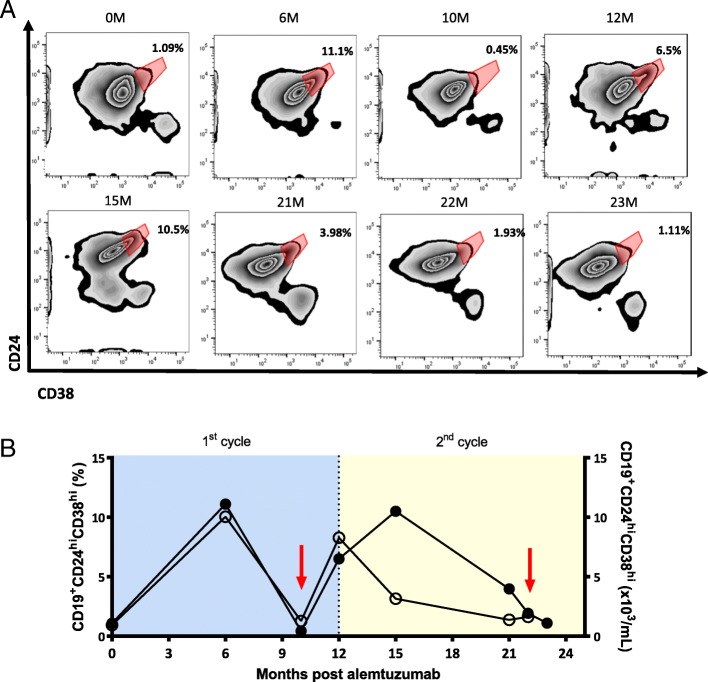
The frequency of CD19^+^CD24^hi^CD38^hi^ cells is decreased during relapse post alemtuzumab treatment. **a** Flow-cytometry plot of expression of CD24 and CD38 in total CD19^+^ B cells. **b** Graph showing the clinical course. The frequency and the absolute number of CD19^+^CD24^hi^CD38^hi^ B cells and infusion of 2 cycles of alemtuzumab are shown. Filled circles indicate the frequency of CD19^+^CD24^hi^CD38^hi^ B cells, and hollow circles indicate the absolute number of CD19^+^CD24^hi^CD38^hi^ B cells. Red arrow indicates relapse

Among 11 patients treated with alemtuzumab, one patient experienced two severe relapses within 2 cycles of alemtuzumab infusion (Fig. 6a, b). During his first attack, overshoot of total B cells was observed while the frequency of CD19^+^CD24^hi^CD38^hi^ cells was remarkably decreased to 0.45% compared to previous follow-up (6 M: 11.1%). 4 months later, the frequency of CD19^+^CD24^hi^CD38^hi^ cells increased to 6.5%. 3 months post second alemtuzumab infusion, the frequency of CD19^+^CD24^hi^CD38^hi^ cells was 10.5%. However, at his second attack, 10 M after second cycle infusion, the frequency of CD19^+^CD24^hi^CD38^hi^ cells was again markedly decreased to 1.93% accompanied by overshooting B cell response. Interestingly, the reduction in the frequency of CDa, b19^+^CD24^hi^CD38^hi^ cells (3.98%) 20 days preceding second attack was observed, which is an immense reduction from previous follow-up (15 M: 10.5%). In contrast to CD19^+^CD24^hi^CD38^hi^ cells, no change in the frequency of CD19^+^PD-L1^hi^ cells was observed during both relapses. The absolute number of CD19^+^CD24^hi^CD38^hi^ cells was also reduced during relapses.

## Discussion

Little is known about the repopulating B cell pool following alemtuzumab. Cases of severely exacerbated central nervous system inflammation in alemtuzumab-treated MS patients have reported B-cell driven pathology, further emphasizing the importance of B cell study in alemtuzumab-treated patients [13–15]. We show that deficiency of CD19^+^CD24^hi^CD38^hi^ cells and CD19^+^PD-L1^hi^ cells in the peripheral blood of relapsing MS patients are restored following alemtuzumab and that B cell distribution shifts towards naïve phenotype. In fact, the frequency of Bregs with disparate regulatory mechanisms exceeded baseline level, which may underlie the long-lasting suppression of disease activity.

Interestingly, both frequency and absolute number of CD19^+^CD24^hi^CD38^hi^ cells are reduced during and prior to relapse in an alemtuzumab-treated patient. CD19^+^CD24^hi^CD38^hi^ cells are known to maintain Tregs and limit the differentiation of T helper 1 (Th1) and T helper 17 (Th17) cells [16]. The deficiency of CD19^+^CD24^hi^CD38^hi^ cells could have a significant impact on the regulation of pathology. Indeed, recent studies have found that transitional B cells are impaired in various immune-related disorders [7, 16–18], although conflicting results were observed in MS. [19, 20] In the early phase of alemtuzumab therapy, CD19^+^ B cells repopulate earlier than CD4+ T cells and immature B cells dominate the repopulated CD19+ B cells [21]; the extensive repopulation of CD19^+^CD24^hi^CD38^hi^ cells following alemtuzumab may contribute to the expansion of Tregs while suppressing differentiation of naïve CD4^+^ T cells into Th1 and Th17 cells and hence, contributing to the efficacy of alemtuzumab.

A recent study described that CD19^+^PD-L1^hi^ cells are capable of suppressing Tfh cell differentiation and expansion through interaction with PD-1 on activated T cells [8]. Interaction causes an increase in signal transducer and activator of transcription 5 expression, a known suppressor of Tfh-cell development and expansion. Since Tfh cells aid germinal center formation and hence, involved in the formation of memory B cells and plasma cells, Tfh cells were thought to have pathogenic role in the B-cell-mediated autoimmune diseases. There has been several reports on Tfh cell involvement in MS. [22–25] Most of all, there has been report that CCR7^+^ ICOS^+^ circulating memory Tfh cells are increased in MS patients during relapse, but decreased in patients during remission [9]. This finding is in line with our results, where CD19^+^PD-L1^hi^ cells are decreased in MS patients during relapse, but restored during remission. Hence, suppression of Tfh cell differentiation and proliferation by CD19^+^PD-L1^hi^ cells may be impaired due to CD19 + PD-L1hi cell deficiency, contributing to enhancement of disease activity.

This study is limited by its small sample size. In addition, we report that the marked reduction of CD19^+^CD24^hi^CD38^hi^ cells during relapse in an alemtuzumab-treated patient was observed in only 2 relapses of a single case. Therefore, in order to decipher the critical role of CD19^+^CD24^hi^CD38^hi^ cells in the long-term disease suppression of MS and in the mechanism of action of alemtuzumab, further longitudinal study with larger number of patients on how CD19^+^CD24^hi^CD38^hi^ cells and other lymphocytes (including Tregs) change during relapse, is required. The extensive expansion of CD19^+^CD24^hi^CD38^hi^ cells was maintained until 9 months post-alemtuzumab, whereas CD19^+^PD-L1^hi^ cells were maintained until the end of the cycle. Further work needs to be established to explain the difference in the results. In addition, a recent study has reported that hyperrepopulation of immature B cells post-alemtuzumab in the absence of adequate regulation by T cells increases the risk of secondary autoimmunity [21]. Due to vast composition of immature B cells and lack of understanding of their function, it remains to be further elucidated which specific immature B cell subset would be responsible for the secondary autoimmunity. Lastly, further functional study on the CD19^+^CD24^hi^CD38^hi^ cells would clarify the mechanism of action of alemtuzumab.

## Conclusion

Our results highlight the preferential reconstitution of Bregs as a possible mechanism of action of alemtuzumab and CD19^+^CD24^hi^CD38^hi^ cells as a potential biomarker for disease activity.

## Abbreviations

Bregs: Regulatory B cells
CD19^+^CD24^hi^CD38^hi^: Transitional B cells
HC: Healthy controls
MS: Multiple sclerosis
MS-relapse: MS patients during relapse
MS-remission: MS patients in remission
PBMC: Peripheral blood mononuclear cells
PD-L1: Programmed death-ligand 1
Tfh: T follicular helper cells
Th1: T helper 1
Th17: T helper 17
Tregs: Regulatory T cells
